# Utilisation and associated socio-demographic factors related to the maternal continuum of care in sub-Saharan Africa: A systematic review and meta-analysis

**DOI:** 10.7189/jogh.14.04180

**Published:** 2024-10-18

**Authors:** Getachew Mullu Kassa, Frezer Abebe Dulume, Robera Olana Fite, Kassahun Alemu, Alemayehu Worku, Lisanu Taddesse, Delayehu Bekele, Getachew Tolera, Grace J Chan, Alemnesh H Mirkuzie

**Affiliations:** 1 Health System and Reproductive Health Research Directorate, Ethiopian Public Health Institute, Addis Ababa, Ethiopia; 2 UNICEF, Addis Ababa, Ethiopia; 3 HaSET Maternal, Neonatal and Child Health Research Program, Addis Ababa, Ethiopia; 4 School of Public Health, College of Health Sciences, Addis Ababa University, Addis Ababa, Ethiopia; 5 Department of Obstetrics and Gynecology, Saint Paul's Hospital Millennium Medical College, Addis Ababa, Ethiopia; 6 Duputy Director, Research and Technology Transfer Directorate, Ethiopian Public Health Institute, Addis Ababa, Ethiopia; 7 Department of Epidemiology, Harvard University T.H. Chan School of Public Health, Boston, Massachusetts, USA; 8 Department of Pediatrics, Boston Children’s Hospital, Harvard Medical School, Boston, Massachusetts, USA; 9 John Snow, Inc. (JSI), Addis Ababa, Ethiopia

## Abstract

**Background:**

Maternal continuum of care (MCC) is the utilisation of maternal health care services, including 4+ antenatal care (ANC) visits, skilled birth attendants (SBAs), and postnatal care (PNC). This systematic review and meta-analysis assessed the pooled proportion of MCC utilisation among women in sub-Saharan Africa (SSA) and its association with selected sociodemographic factors.

**Methods:**

We identified keywords and MeSH terms related to the condition (MCC), the context (SSA), and population (women with history of childbirth) to search for published or unpublished observational studies. We used the Joanna Briggs Institute tool to extract data and the Newcastle Ottawa Scale for quality assessment. Meta-analysis was used to compute pooled estimates (MCC utilisation and odds ratio (OR) associates) with 95% confidence intervals (CI) using Stata 17.

**Results:**

Of 45 402 studies identified, we included 23 involving 320 353 women. The pooled estimate of MCC utilisation across SSA was 18.72% (95% CI = 14.51, 22.93), showing a significant increase (*P* < 0.05) from 2015 to 2022. Southern Africa had the highest MCC utilisation (38%; 95% CI = 36.59, 39.41), while East Africa had the lowest (17.5%; 95% CI = 12.22, 22.75). Maternal continuum of care utilisation was associated with maternal age 25–34 years (pooled odds ratio (POR) = 1.27), urban residence (POR = 2.69), richer/richest wealth status (POR = 1.68), as well as higher level of education and employment (POR = 1.32).

**Conclusions:**

MCC utilisation in SSA remains low, with significant variation across the sub-regions and sociodemographic strata. Context-specific interventions targeting identified factors are essential to enhance MCC utilisation in SSA.

**Registration:**

PROSPERO: CRD42021272708.

A high maternal mortality rate (MMR) is one of the major health and development challenges, particularly in sub-Saharan Africa (SSA). Approximately 60% of maternal deaths occur during childbirth and the immediate postnatal period [1]. Although there are numerous reasons for maternal deaths, the lack of maternal health care services during pregnancy, childbirth, and the postnatal period appears to be a major factor [2–4].

The maternal continuum of care (MCC) is a key strategy for reducing maternal and neonatal morbidity and mortality globally [3,4]. It is the utilisation of a continuum of care in relation to maternal health care services, including four or more antenatal care (ANC) visits, skilled birth attendants (SBAs), and postnatal care (PNC) [5].

Current evidence indicates that the MCC is associated with maternal mortality [6], as well as evidence that it is a critical intervention for improving the utilisation of child health services [7]. Maternal continuum of care is viewed as being essential to meet national and global health-related targets focusing on improving the health and survival of women, newborns, and children [8]. Most countries in SSA have incorporated universal access approaches to reproductive health, including MCC as one of the main strategies to reduce MMR and child mortality [9]. Kenya reported 34% coverage of MCC [10], Tanzania 10% [11], and Ghana 7.9% [12].

Previous studies have identified factors that were associated with MCC utilisation [13–21]. However, the literature on MCC utilisation in SSA shows inconsistent and inconclusive findings. Estimating the overall regional and sub-regional distributions of MCC utilisation and associated factors could provide essential evidence to guide policies and programmes. Therefore, this systematic review and meta-analysis (SRMA) was conducted to assess the pooled proportion of MCC utilisation and its association with selected sociodemographic factors in SSA.

## METHODS

### Protocol and registration

This review was conducted according to a priori registered protocol on PROSPERO: CRD42021272708. The SRMA study followed the 2015 Preferred Reporting Items for Systematic Reviews and Meta-analyses Protocol (PRISMA-P) [22,23]. We also followed the PRISMA 2020 statement guideline [24] to report the study findings. The data are presented in Table S1 in the **Online Supplementary Document**.

### Eligibility criteria

Our review included studies using a combination of condition, context, and population (CoCoPop) criteria. MCC was defined as the continuity of care throughout pregnancy, childbirth, and postnatal care [25]. The primary outcome of this study was the utilisation of MCC (condition), a composite variable score defined as the score of use of four or more ANC visits, SBA, and one or more PNC [3,7,26]. Complete utilisation of MCC was defined as the use of all these maternal health care services. However, participants who did not use at least one type of these services (e.g. fewer than four ANC visits, or no SBA, or no PNC) were defined as having incomplete utilisation of MCC. This review also examined the relationship of sociodemographic factors as exposure variables with MCC utilisation. We included maternal age, marital status, residence, wealth status, educational status, and employment status. We only included studies that were conducted in SSA countries (context). The list of SSA countries was retrieved from the World Bank Group website [27]. All studies that enrolled pregnant women, postnatal women or women who gave birth at least once (population) were included. The current review included studies published from 1 January 2005 through the date of the last database search, 18 June 2022. We selected 2005 considering that MCC was one of the major themes in the 2005 World Health Report and the Lancet Neonatal Survival Series [28,29]. The subsequent emphasis on MCC at the World Health Assembly in 2007 further underscores its importance in global health discussions during this period [5]. Therefore, the year 2005 was selected as it marked the beginning of a significant shift in focus towards the MCC in major health reports and series. Observational studies, including cross-sectional, case-control, and cohort studies, either published or reported in English, were included in this review. Both published and grey literature were considered.

### Exclusion criteria

We excluded case studies, editorials in journals, and studies with insufficient data on the utilisation of MCC from the study. Furthermore, studies that reported separate service utilisation (either ANC, SBA, or PNC) without reporting MCC outcome were also excluded from the study.

### Search strategy and sources

Published studies were searched in major databases including PubMed/MEDLINE, Embase, Web of Science, CINAHL, and African Journals Online (AJOL). We also searched for unpublished studies, thesis, and dissertations in grey literature sources such as Google Scholar, Google hand search, and institutional repository websites of universities in SSA. We also screened references to retrieve additional studies that could be included in this review. A combination of different search terms, both text words and Medical Subject Headings (MeSH) terms, was developed by using the CoCoPop criteria in a logic grid. The search terms were combined using the Boolean operators ‘OR’ or ‘AND’ to either narrow or broaden the search strategy. The search history in PubMed database is presented in Table S2 in the **Online Supplementary Document**. All retrieved studies from different databases were then exported to EndNote Version 20 [30] for article screening and management. The search and study screening (title, abstract, and full-text screening) were conducted by two independent researchers using the prior eligibility criteria.

### Data extraction

Two authors extracted data independently using a tool developed by the Joanna Briggs Institute (JBI) for prevalence studies [31] considering the primary outcome of the study. Then, the extracted data were compared, and discrepancies resolved by consensus or by involving a third author. The authors also developed a data extraction checklist for the study’s exposure variables. This checklist included information on author name, title, data collection period, publication year, study design, study setting, geographic region, study population, sample size, and response rate. It also covered the definition of MCC, the tool used to measure MCC, the percentage of complete MCC utilisation, raw data for the number of cases, total sample size, and other characteristics of the study population. To identify the factors associated with the MCC, we extracted raw data for each category of the outcome and exposure variables using Microsoft Excel. Additionally, we contacted the authors of primary studies to obtain any missing data relevant to the current review and meta-analysis.

### Quality appraisal

The quality of included studies was assessed using the Newcastle Ottawa Scale (NOS) of quality assessment from previously conducted studies [32,33]. The quality assessment was done by two authors independently, and any discrepancies were resolved through discussion. The NOS contains three major criteria to assess the quality of the studies: selection, comparability, and outcome. Under the selection criteria, issues such as representativeness of the sample, sample size, non-response, and ascertainment of the exposure (risk factors) were included. The comparability criteria included assessment of the study participants in different outcome groups and if confounding factors were controlled or not. The outcome criteria focused on how the outcome was assessed and the use of appropriate statistical tests. A score of 9–10 points was classified as very good quality, 7–8 points as good quality, 5–6 points as satisfactory quality, and 0–4 points as unsatisfactory studies.

### Data processing and analysis

The extracted data from individual studies were exported to Stata software (version 17; StataCorp, College Station, TX) for meta-analysis. The analysis was conducted using the random-effects model to minimise heterogeneity among the included studies and to account for both random variability and variability in effects among the studies [34,35]. The pooled meta-analysis result for MCC utilisation and associated factors was presented using forest plots. Pooled odds ratio (POR) with a 95% confidence interval (CI) was computed using a meta-analysis method to determine the association between MCC utilisation and sociodemographic factors.

### Heterogeneity and meta-regression

The heterogeneity of the studies was assessed using Cochran's Q, τ^2^, and *I*^2^ statistics. A *P*-value less than 0.05 was used to declare significant heterogeneity among the included studies. The *I*^2^ statistics described the percentage of total variation across studies due to heterogeneity rather than chance. A result of zero percent and larger values indicated no heterogeneity and increasing heterogeneity, respectively [36]. Additionally, *I*^2^ statistical results of 25% were used to assign heterogeneity as low, 50% as moderate, and 75% as high [34]. Moreover, if the heterogeneity test was found statistically significant, we conducted a meta-regression analysis to determine the study-level covariates that could explain the heterogeneity of included studies [37,38]. The meta-regression analysis was conducted using several study-related characteristics including publication year, study area/country, sub-regional category, sample size, and quality of the study.

### Sensitivity and subgroup analyses

To ensure the robustness of outcomes of the meta-analysis and assess the influence of a single study on the overall meta-analysis, a sensitivity analysis was conducted [35,39]. Furthermore, a series of subgroup analysis was conducted to estimate the effect sizes, including pooled utilisation level of MCC and pooled ORs, of different study variables including study period, publication year, study setting, geographic region, sub-regional category, quality of the studies, and other study-related characteristics.

### Publication bias

The presence of publication bias among the included studies was assessed using visual inspection of the funnel plot and Egger’s regression symmetry test [40–42]. Egger’s test *P*-value of less than 0.05 was used to declare statistically significant publication bias. For significant publication bias, Duval and Tweedie's nonparametric trim and fill analysis using the random-effect analysis was conducted [35,43].

## RESULTS

### Study selection

**Figure 1:**
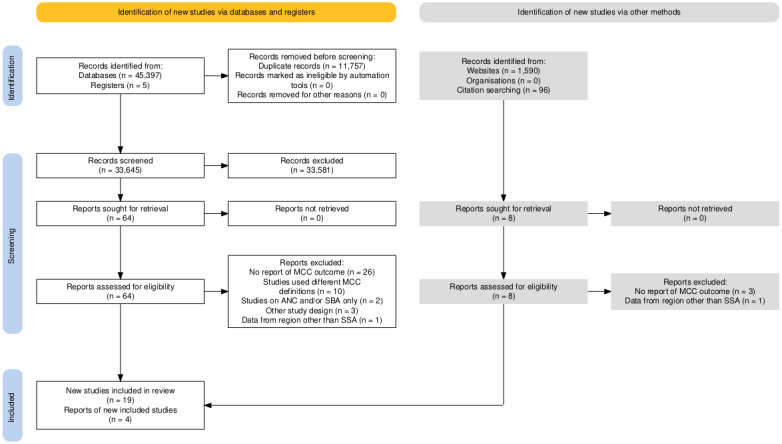
Flow diagram of the article search and selection for the systematic review and meta-analysis of the maternal continuum of care utilisation and its association with sociodemographic factors in sub-Saharan Africa, 2015–2022. ANC – antenatal care, MCC – maternal continuum of care, SBA – skilled birth attendance, SSA – sub-Saharan Africa.

A total of 45 402 studies were identified via databases and registers and 1686 studies identified via other methods. Duplicate articles were removed (11 757) and the remaining articles (33 581) were screened by title and abstract review. A total of 72 articles (64 articles retrieved from databases and registers and eight articles from other sources) were assessed for eligibility in the full-text screen. We excluded 29 articles that did not report the MCC utilisation outcome, 10 because of different definitions of MCC (e.g. some studies included 1 ANC utilisation instead of 4 ANCs), three focusing only on PNC, two focusing only on ANC and/or SBA, three not using observational study designs, and two from non-SSA countries. Thus, we included 23 articles in this review (**Figure 1**).

### Characteristics of included studies

Of the included 23 studies [3,11,14,16–19,26,44–58], 12 were conducted in Ethiopia [3,14,17–19,26,47,50–54], one in Ghana [56], two in Tanzania [11,58], and two in Uganda [44,57]. Additionally, studies from Benin, Guinea, and Zambia were included. Three studies [16,49,55] were conducted across multiple countries. Among the included studies, five were published in 2022 [14,16,17,44,45], five in 2021 [18,19,47–49], five in 2020 [3,26,50–52], and the remaining six articles were published between 2015 and 2019 [11,54–58]. The sample size ranged from 223 in a study conducted in Benin [45] to 225 135 in a multi-country study conducted in SSA [16]. Overall, a total of 320 353 women were included in this systematic review and meta-analysis. The study population mainly consisted of women who had given birth in the last year or in the last two years before the date of data collection for each study (**Table 1**).

**Table 1:** Summary characteristics of studies included in the systematic review and meta-analysis of MCC utilisation and its association with sociodemographic factors in sub-Saharan Africa, 2015–2022

Author, year	Study area, country	Study design	Study population	Study period	Sample size	Response rate (n)
Tiruneh et al., 2022	Amhara, Oromia, SNNP, and Tigray regions, Ethiopia	Cross-sectional	Women who had live births in the last 12 mo preceding the survey	October–November 2017	2724	2724
Alamneh et al., 2022	Ethiopia	Ethiopian DHS	All reproductive aged women who were booked for ANC service and giving birth in the selected EAs within 5 y before the 2016 survey	2016	4772	4772
Sserwanja et al., 2022	Uganda	Uganda DHS	Women aged 15–49 y who had a live birth within five years preceding the survey	June–December 2016	10 152	10152
Gryseels et al., 2022	Selected villages in the Atlantique region in the South of Benin	Benin DHS	Women included in BDHS from 2017 to 2018	BDHS 2017–2018 and qualitative study between 2018 and 2019	223	223
Alem et al., 2022	DHS conducted in SSA from 2010 to 2018*	DHSs	Women who gave birth in the last two years	DHS data 2010–2018	225 135	225 135
Tizazu et al., 2021	Debre Berhan Town, Amhara, Ethiopia	Cross-sectional	Women who gave birth in the last one year	17 February–15 March 2020	659	647
Sserwanja et al., 2021	10 provinces, Zambia	Cross-sectional	Women of reproductive age (15–49 y) who had given birth in the last five years preceding the survey	18 July 2018–24 January 2019	7325	7325
Sertsewold et al., 2021	Eight Kebeles, Siyadebirena Wayu district, Ethiopia	Cross-sectional	Women who gave birth in the last one year	1 February–10 March 2020	620	614
Dadi et al., 2021	62 woredas in nine regions, Ethiopia	Cross-sectional	Women who gave birth in the last two years	March–May 2019	1431	1431
Camara et al., 2021	Guinea	Guinea DHS	Women who gave birth in the last two years	March–June 2018	3018	3018
Arunda et al., 2021	Countrywide, Kenya and Uganda	DHSs	Most recent live births of mothers within 1 to 59 mo prior to the 2014 to 2016 DHS	2014–2016 DHS	24 502	24 502
Shitie et al., 2020	Enemay district, Ethiopia	Cross-sectional	Women who gave birth in the last one year	25 February–March 10 2019	651	621
Muluneh et al., 2020	Ethiopia DHS	DHS	Currently pregnant women	2016	4693	4693
Haile et al., 2020	Arba Minch Zuria woreda, Gamo, Ethiopia	Cross-sectional	Pregnant women who started ANC services	15 February–15 March 2019	438	432
Emiru et al., 2020	West Gojjam Zone, Amhara, Ethiopia	Cross-sectional	Women who gave birth in the last one year	June 2018	1337	1281
Atnafu et al., 2020	North Gondar Zone, Amhara, Ethiopia	Cross-sectional	Women who gave birth in the past five years	1 May–29 June 2019	583	565
Haile et al., 2019	Arba Minch Zuria Woreda, Gamo Zone, Ethiopia	Cross-sectional	Women gave birth after booked for ANC in health facilities and were at or after six weeks of childbirth	15 February–March 2019	595	583
Chaka et al., 2019	Ethiopia	Ethiopian DHS	Women who had reported that they have received at least 4 ANC visits and SBA	2016 EDHS	7590	7590
Mohan et al., 2017	Four districts of Tanzania’s Morogoro region, Tanzania	Cross-sectional	women who delivered in the preceding 2 to 14 mo	2011	1968	1869
Singh et al., 2016	Ethiopia, Malawi, Rwanda, Senegal, Tanzania, and Uganda	Recent DHSs	Women who gave birth in the last one year	Recent DHS from 2010 onwards	18 036	18 036
Yeji et al., 2015	Three regions of Ghana	Cross-sectional	Reproductive age women	January 2011 and April 2013	1500	1497
Rutaremwa et al., 2015	Uganda	Uganda DHS	Women who gave birth in the last one year	2011 UDHS	1764	1728
Larsen et al., 2015	Rufiji, Ulanga, and Kilombero districts, Tanzania	Cross-sectional	Women who gave birth in the last two years	May and July 2011	915	915

ANC – antenatal care, BDHS – Benin Demographic and Health Survey, DHS – Demographic and Health Survey, EA – enumeration areas, EDHS – Ethiopian Demographic and Health Survey, HDSS – Health Demographic Surveillance Sites, SBA – skilled birth attendance, SNNP – Southern Nation Nationalities People, SSA – sub-Saharan Africa, UDHS – Uganda Demographic and Health Survey

*Lesotho, Namibia, South Africa, Zimbabwe, Angola, DR Congo, Congo, Cameroon, Gabon, Chad, Burundi, Ethiopia, Kenya, Malawi, Rwanda, Tanzania, Comoros, Uganda, Zambia, Burkina-Faso, Benin, Cote devoir, Ghana, Gambia, Guinea, Liberia, Mali, Nigeria, Niger, Sierra Leone, Senegal, Togo.

### Quality assessment

The results of the NOS scores for the 23 included studies categorised 11 studies as ‘very good quality’ and 12 as ‘good quality’. The mean score ± standard deviation (SD) for the selection criteria was 4.35 ± 0.63 out of 5, for comparability criteria was 1.3 ± 0.56 out of 2, and for outcome criteria was 2.22 ± 0.41 out of 3. Overall, the mean quality score and SD out of 10 points was 8.4 ± 0.77 (**Table 2**).

**Table 2:** Quality Score assessment result using the Newcastle Ottawa Scale for a systematic review and meta-analysis of the maternal continuum of care utilisation and its association with sociodemographic factors in sub-Saharan Africa, 2015 to 2022

Author, year	Country	Selection					Comparability			Outcome			Overall Score (10)	Quality Category
		**Representativeness of the sample**	**Sample size**	**Non-respondents**	**Ascertainment of the exposure (risk factor)**	**Total score (5)**	**Study controls for most important factor**	**Study controls for additional factors**	**Total Score (2)**	**Assessment of outcome**	**Statistical test**	**Total score (3)**		
Tiruneh et al., 2022	Ethiopia	1	1	1	1	4	1	1	2	1	1	2	8	Good
Alamneh et al., 2022		1	1	1	2	5	1	1	2	1	1	2	9	Very good
Sserwanja et al., 2022	Uganda	1	1	1	2	5	1	1	2	2	1	3	10	Very good
Gryseels et al., 2022	Benin	1	1	1	2	5	0	0	0	2	1	3	8	Good
Alem et al., 2022	33 SSA countries	1	1	0	2	4	1	1	2	2	1	3	9	Very good
Tizazu et al., 2021	Ethiopia	1	1	0	2	4	1	1	2	1	1	2	8	Good
Sserwanja et al., 2021	Zambia	1	1	0	2	4	1	1	2	2	1	3	9	Very good
Sertsewold et al., 2021	Ethiopia	1	1	0	1	3	1	1	2	1	1	2	7	Good
Dadi et al., 2021	Ethiopia	1	1	1	1	4	1	1	2	1	1	2	8	Good
Camara et al., 2021	Guinea	1	1	1	1	4	1	1	2	1	1	2	8	Good
Arunda et al., 2021	Kenya and Uganda	1	1	1	2	5	1	1	2	1	1	2	9	Very good
Shitie et al., 2020	Ethiopia	1	1	1	1	4	1	1	2	1	1	2	8	Good
Muluneh et al., 2020		1	1	1	2	5	1	1	2	2	1	3	10	Very good
Haile et al., 2020		1	1	1	2	5	1	1	2	1	1	2	9	Very good
Emiru et al., 2020		1	1	1	2	5	1	1	2	1	1	2	9	Very good
Atnafu et al., 2020		1	1	1	1	4	1	1	2	1	1	2	8	Good
Haile et al., 2019		1	1	1	1	4	1	1	2	1	1	2	8	Good
Chaka et al., 2019		1	1	1	2	5	1	1	2	1	1	2	9	Very good
Mohan et al., 2017	Tanzania	1	1	1	2	5	0	0	0	1	1	2	7	Good
Singh et al., 2016	Ethiopia, Malawi, Rwanda, Senegal, Tanzania, and Uganda	1	1	1	1	4	1	1	2	1	1	2	8	Good
Yeji et al., 2015	Ghana	1	1	1	2	5	1	1	2	1	1	2	9	Very good
Rutaremwa et al., 2015	Uganda	1	1	1	2	5	1	1	2	1	1	2	9	Very good
Larsen et al., 2015	Tanzania	1	1	1	0	3	1	1	2	1	1	2	7	Good

SSA – sub-Saharan Africa

### Overall MCC utilisation

**Figure 2:**
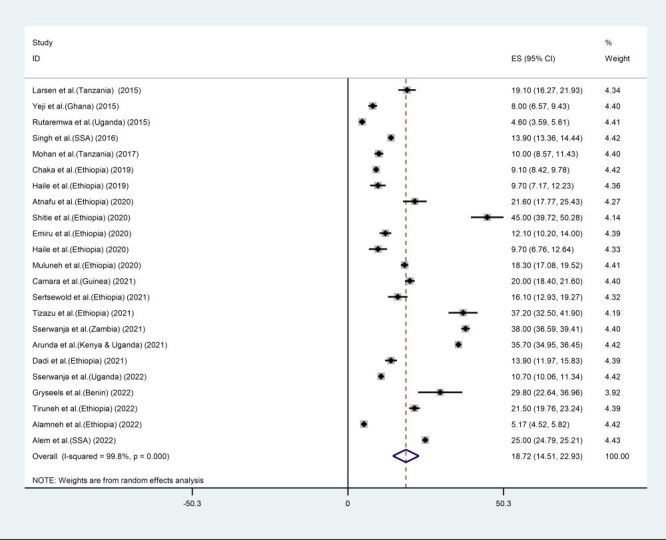
The pooled proportion of maternal continuum of care utilisation in sub-Saharan Africa, 2015–2022. CI – confidence interval, ES – effect size, SSA – sub-Saharan Africa.

We estimated the overall proportion of MCC utilisation using the included 23 studies. The proportion of MCC utilisation ranged from 4.6% in a study conducted in Uganda using the 2011 Demographic and Health Survey (DHS) [57] to 45% in Ethiopia in 2019 [50]. The pooled proportion of MCC utilisation was 18.72% (95% CI = 14.51, 22.93) (**Figure 2**).

### Subgroup analyses

The analysis of the proportion of MCC utilisation by sub-regions in Africa showed higher service utilisation in Southern Africa (38%), although the finding is from only one large study [46]. In West Africa, using three studies, the proportion of MCC utilisation was 18.75%, and in East Africa, using 17 studies, it was 17.49%. The proportion of MCC utilisation in studies categorised as good quality was 20.81% (95% CI = 17.4, 24.23), whereas for studies categorised as very good quality, it was 16.04% (95% CI = 9.33, 22.76) (**Table 3**).

**Table 3:** Subgroup analysis by selected study characteristics to determine the proportion of maternal continuum of care utilisation in sub-Saharan Africa, 2015 to 2022

Subgroup analysis	Number of studies	Sample size	Proportion (95% CI)	Heterogeneity		
				**τ^2^**	***I^2^***	***P-*value**
Country						
*Ethiopia*	12	25 953	17.87 (13.80, 21.90)	48.57	98.8	<0.001
*Uganda*	2	11 880	7.66 (1.69, 13.64)	18.40	99.0	<0.001
*Tanzania*	2	2784	14.50 (5.55, 23.40)	40.10	96.8	<0.001
*Three or More African countries*	2	243 171	19.45 (8.58, 30.33)	61.60	99.9	<0.001
*Ghana*	1	1497	8.00 (6.60, 9.40)	0.00	-	-
*Guinea*	1	3018	20.00 (18.40, 21.60)	0.00	-	-
*Benin*	1	223	29.80 (22.64, 36.96)	0.00	-	-
*Zambia*	1	7325	38.00 (36.60, 39.40)	0.00	-	-
*Kenya and Uganda*	1	24 502	35.70 (34.95, 36.50)	0.00	-	-
Sub-region						
*East Africa*	17	65 394	17.50 (12.22, 22.75)	120.90	99.7	<0.001
*West Africa*	3	4741	18.75 (8.50, 28.99)	77.50	98.6	<0.001
*Southern Africa*	1	7325	38.00 (36.59, 39.41)	0.00	-	-
SSA*	2	243 171	19.45 (8.59, 30.33)	61.60	99.9	<0.001
Quality score of studies						
*Good*	12	31 423	20.81 (17.4, 24.23)	33.40	97.4	<0.001
*Very good*	11	289 208	16.04 (9.33, 22.76)	128.60	99.9	<0.001
Year published						
*2015*	3	4140	10.40 (4.24, 16.56)	28.68	97.9	<0.01
*2016*	1	18 036	13.90 (13.36, 14.44)	0.00	-	-
*2017*	1	1869	10.00 (8.57, 11.43)	0.00	-	-
*2019*	2	8173	9.14 (8.48, 9.80)	0.00	0	0.653
*2020*	5	7592	20.93 (13.93, 27.93)	60.86	97.6	<0.01
*2021*	6	37 537	26.79 (18.29,35.28)	111.00	99.4	<0.01
*2022*	5	243 006	18.22 (8.00, 28.43)	133.00	99.9	<0.01
Overall	23	320 631	18.72 (14.51, 22.93)	104.20	99.8	<0.001

CI – confidence interval, SSA – sub-Saharan Africa

*Studies that included countries in more than one sub-regional category.

**Figure 3:**
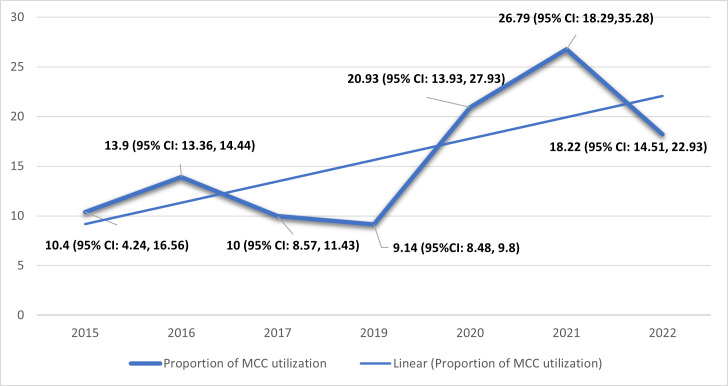
Trend of pooled maternal continuum of care utilisation in sub-Saharan Africa, 2015–2022. CI – confidence interval, MCC – maternal continuum of care.

The trend of MCC utilisation, assessed using the Extended Mantel-Haenszel χ^2^ for linear trend analysis, showed a significant increase from 2015 to 2022, with a *P*-value <0.01 **(****Figure 3**).

### Factors associated with MCC utilisation

To identify the sociodemographic factors associated with MCC utilisation in SSA, we included maternal age, marital status, residence, wealth status, educational status, and employment status. The pooled meta-analysis to estimate OR associations showed that all variables, except marital status, were significantly associated with MCC utilisation.

To identify the association between maternal age and MCC utilisation, we included a total of 10 studies and categorised maternal age as reported by each study. Seven studies used age categories of 15 to 24 years, 25 to 34 years, and 35 to 49 years, while the remaining three studies reported age categories of <19 years, 20 to 34, and 35 to 49 years. Accordingly, when comparing women aged 15 to 24 with those aged 35 to 49, four studies showed that older women are less likely to utilise MCC compared to younger mothers. A contrasting finding was observed in one study, while two studies showed a non-significant difference in the odds of MCC utilisation between younger and older mothers. The pooled meta-analysis comparing the association between the two age groups showed a non-significant difference in the odds of MCC utilisation (POR = 0.88; 95% CI = 0.72, 1.09)). Similarly, a non-significant difference was observed in the odds of MCC utilisation when comparing women aged 15 to 24 with women aged 25 to 34 (POR = 1.01; 95% CI = 0.94, 1.09)), pooling the findings from seven studies.

However, the pooled meta-analysis comparing women aged 25 to 34 with those aged 35 to 49 showed a statistically significant variation in MCC utilisation. The odds of MCC utilisation among women aged 25 to 34 were 1.27 times higher compared to women aged 35 to 49 (POR = 1.27; 95% CI = 1.13, 1.43). Six of the studies reported similar findings, while only one study showed a non-significant difference in the odds of MCC utilisation between the two groups. Additionally, subgroup meta-analysis comparing women aged <19 with women aged 20 to 34 and 35 to 49 years, as well as women aged 20 to 34 with those aged 35 to 49 showed a non-significant difference in the odds of MCC utilisation (**Table 4**).

**Table 4:** Results of the pooled meta-analysis for the socio-demographic factors associated with the maternal continuum of care in sub-Saharan Africa, 2015 to 2022

Women Characteristics	Categories	Reference Category	Countries	Year of Publication	Number of Studies	Total Sample Size	POR (95% CI)	Heterogeneity			
								**τ^2^ (%)**	***I* ^2^**		***P*-value**
Age	<19 vs. 20 to 34 y	<19 y	Ethiopia (2), Uganda	2015–2022	3	7676	1.18 (0.88, 1.57)	0.00	0.0	0.828	
	20 to 34 vs. 35 to 49 y	35 to 49 y	Ethiopia (2), Uganda	2015–2022	3	8821	1.01 (0.84, 1.20)	0.00	0.0	0.457	
	15 to 24 vs. 25 to 34 y	15 to 24 y	Ethiopia (3), Uganda, Kenya & Uganda, SSA. Zambia	2020–2022	7	205 055	1.01 (0.94, 1.09)	0.50	67.0	0.006	
	25 to 34 vs. 35 to 49 y	35 to 49 y	Ethiopia (3), Uganda, Kenya & Uganda, SSA. Zambia	2020–2022	7	187 244	1.27 (1.13, 1.43)*	1.37	80.0	<0.001	
	<19 vs. 35 to 49 y	<19 y	Ethiopia (2), Uganda	2015–2022	3	2421	1.13 (0.81, 1.57)	0.00	0.0	0.765	
	15 to 24 vs. 35 to 49 y	15 to 24 y	Ethiopia (3), Uganda, Kenya & Uganda, Zambia, SSA	2020–2022	7	145 407	0.88 (0.72, 1.09)	5.33	93.2	<0.001	
Marital status	Married vs. unmarried/single/divorced/widowed)	unmarried/single/divorced/widowed women	Ethiopia (2), Zambia, Uganda, Kenya & Uganda, SSA	2015–2022	6	274 235	0.90 (0.66, 1.21)	11.13	97.3	<0.001	
Place of residence	Urban vs. rural	Rural residence	Ethiopia (6), Uganda (2), Kenya & Uganda, SSA, Zambia	2015–2022	11	276 871	2.69 (2.01, 3.62)*	21.66	98.5	<0.001	
Wealth status	Richer/richest vs. poorer/poorest wealth status	Poorer/poorest wealth status	Ethiopia (2), Uganda (2), Kenya & Uganda, SSA, Zambia	2015–2022	7	276 338	1.68 (1.30, 2.16)*	10.16	97.8	<0.001	
Employment status	Employed vs. not employed	Not employed	Ethiopia (3), Uganda, SSA, Zambia	2019–2022	6	247 967	1.32 (1.10, 1.60)*	3.61	90.3	<0.001	

SSA – sub-Saharan Africa, CI – confidence interval, POR – pooled odds ratio

*Significant factors.

Six studies were included to determine the association between the marital status of women and MCC utilisation. Except for one study [16] that showed lower odds of MCC utilisation among married women compared to unmarried/single/divorced/widowed women, and another study [46] that showed higher odds, the other four studies showed non-significant difference between the two groups. Overall, the pooled meta-analysis showed a non-significant difference in the odds of MCC utilisation between married and unmarried/single/divorced/widowed women (POR = 0.9; 95% CI = 0.66, 1.21). Similar to other factors, the meta-analysis used a random effect model due to significant heterogeneity among the included studies (*P* < 0.001) (**Table 4**).

Eleven studies were included to determine the association between place of residence and MCC utilisation. Except for one study [57] that showed lower odds of MCC utilisation, all other studies in this analysis showed higher odds of MCC utilisation among urban compared to rural resident women. The final pooled meta-analysis also showed that urban resident women had 2.69 times higher odds of MCC utilisation compared to rural residents (POR = 2.69; 95% CI = 2.01, 3.62) (**Table 4**)).

Seven studies were included to determine the association between wealth status and MCC utilisation. Six of the included studies showed significantly higher odds of MCC utilisation among richer/richest compared to poorer/poorest women, whereas one study showed lower odds. Overall, the odds of MCC utilisation were higher among richer/richest women compared to poorer/poorest women (POR = 1.68; 95% CI = 1.3, 2.16) (**Table 4**).

Six studies were included to determine the association between women’s employment status and MCC utilisation. Five of the studies showed a significant increase in the odds of MCC utilisation among employed women compared to unemployed women. Using data from all six studies, the pooled meta-analysis showed that the odds of MCC utilisation were 1.32 times higher among employed women compared to unemployed women (POR = 1.32; 95% CI = 1.1, 1.6) (**Table 4**).

**Figure 4:**
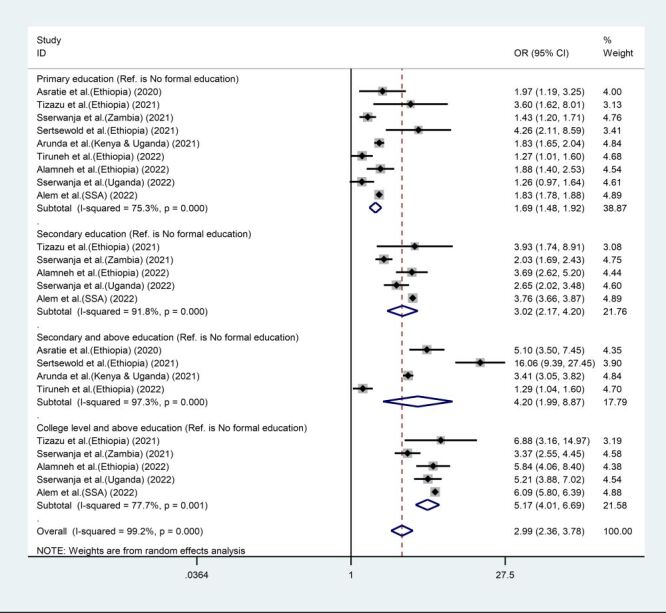
Forest plot showing the odds of maternal continuum of care utilisation among women’s level of education compared to women who had no formal education in sub-Saharan Africa, 2005–2022. CI – confidence interval, OR – odds ratio, SSA – sub-Saharan Africa.

Nine studies were included to determine the association between educational status and MCC utilisation. We used the ‘no formal education’ category as a reference for separate subgroup analyses of primary education, secondary education, secondary and above level of education, and college and above level of education. Accordingly, for all categories, there was a significant increase in the odds of MCC utilisation among primary-educated women (1.69 times), secondary-educated women (3.02 times), secondary and above level of education (4.2 times), and college and above level of education (5.17 times) compared to women with no formal education. Furthermore, the pooled odds ratio increased with higher levels of education (**Figure 4**).

### Heterogeneity and meta-regression

The heterogeneity test using *I*^2^ statistics and the corresponding *P*-value indicated statistically significant heterogeneity among the included studies. We conducted a meta-regression analysis to explore potential sources of this heterogeneity, incorporating covariates such as publication year, country, quality score, and sample size. However, could the analysis did not identify the sources of heterogeneity (*P* > 0.05). This suggests that factors contributing to the observed heterogeneity in the pooled proportion of MCC could be from variables not included in our current analysis. These could include variations in cultural beliefs and geographic accessibility among study participants, variables not covered in this review (**Table 5**).

**Table 5:** Meta-regression analysis of the different study-level covariates explaining possible sources of heterogeneity for meta-analysis of the proportion of Maternal Continuum of Care utilisation in sub-Saharan Africa, 2005 to 2022

Study characteristics	Number of studies	Coefficient	SE	*P*-value
Publication year	23	0.32	1.91	0.870
Quality score	23	−4.93	4.41	0.287
Sample size	23	<0.01	<0.01	0.528
By country				
*Benin*	1	14.70	21.20	0.502
*Ethiopia*	12	9.15	14.20	0.533
*Guinea*	1	5.10	19.70	0.802
*Kenya and Uganda*	1	27.93	18.70	0.165
*SSA*	2	−27.90	20.40	0.199
*Uganda*	2	1.89	19.45	0.135
Sub-regional category				
*East Africa*	17	−31.40	19.40	0.135
*West Africa*	3	−27.70	19.40	0.182

SE – standard error, SSA – sub-Saharan Africa

Sensitivity analysis was employed to assess the impact of individual studies included in the overall pooled meta-analysis. The results indicated that the estimates derived from sensitivity analysis fell within the confidence interval of the pooled meta-analysis, affirming robustness of the meta-analysis findings.

### Publication bias

**Figure 5:**
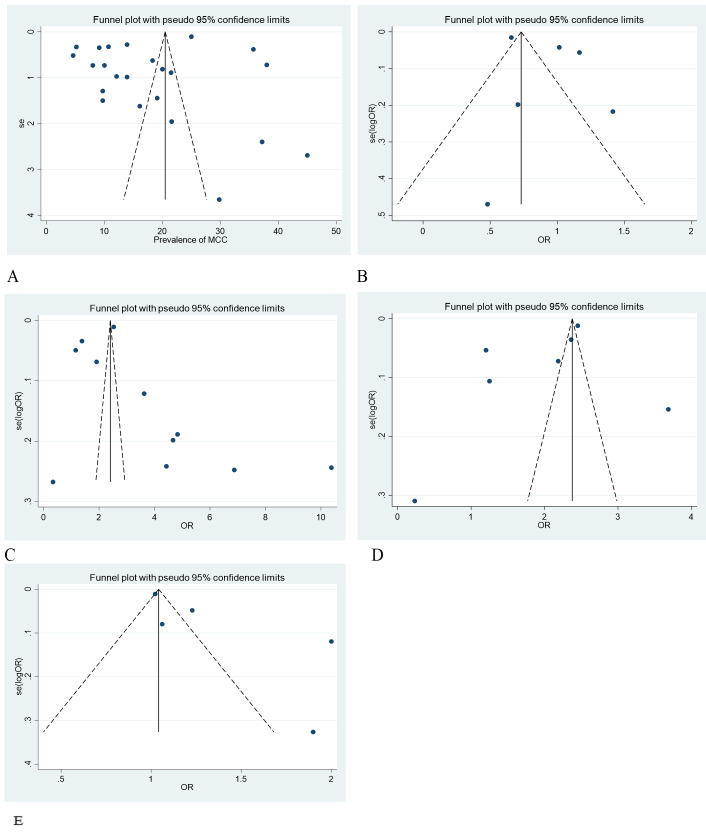
Funnel plots showing the publication bias status of studies included in different outcomes of the study, 2005–2022. **Panel A.** Proportion of maternal continuum of care utilisation (egger’s test *P*-value = 0.168). **Panel B.** Marital status (egger’s test *P*-value = 0.235). **Panel C.** Residence (egger’s test *P*-value = 0.537). **Panel D.** Wealth status (egger’s test *P*-value = 0.238). **Panel E.** Employment status (egger’s test *P*-value = 0.052). MCC – maternal continuum of care, OR – odds ratio, SE – standard error.

We utilised funnel plots and Egger's test for small-study effects, and found no significant publication bias across all outcomes (*P* > 0.05). Therefore, we did not proceed with Duval and Tweedie's trim and fill analysis (**Figure 5****,** Panels A–E).

## DISCUSSION

This SRMA revealed that only 18.72% of women in SSA utilise complete MCC services, highlighting low coverage. A higher MCC service utilisation was observed in Southern Africa, followed by West Africa, with the lowest in Eastern Africa. The sociodemographic factors that were associated with MCC utilisation included maternal age, urban residence, higher wealth status, educational status, and employment status.

Our study identified that approximately one-fifth of women completed the continuum of maternal health care from pregnancy to the postpartum period. This rate is lower compared to studies from countries outside SSA; for instance, MCC utilisation rates were reported as 60% in Cambodia and 46% in Nepal [59,60]. Utilisation of maternal health services brings multidimensional benefits for women and children. In Ethiopia, access to childhood immunisation was four times higher among women who utilised ANC and 1.6 times higher among those who delivered at health facilities [61]. Similar findings were observed in Benin, where incomplete lack of assistance from SBA, and absence of PNC visits were associated with lower odds of full immunisation (adjusted odds ratio (AOR) = 0.11, AOR = 0.55, AOR = 0.11, respectively [62]. Moreover, the provision of complete MCC services has been linked to significant reductions in neonatal, perinatal, and maternal mortality [63]. Therefore, interventions aimed at enhancing MCC utilisation are crucial to mitigate maternal and child mortality.

Our study also identified a significant increase in the MCC utilisation between 2015 and 2022. This positive trend suggests that efforts aimed at improving maternal health care access and delivery across SSA have likely resulted in substantial improvements in MCC utilisation. Factors such as increased awareness, enhanced health care infrastructure, and targeted interventions may contribute to this trend [64]. The rise in MCC utilisation may have also contributed to improvements in maternal and child health outcomes [65]. However, further research is needed to identify specific drivers of this increase and potential disparities in MCC utilisation trends across different sub-regions and health care systems in SSA.

The current study identified variations in MCC utilisation across the different sub-regions in SSA: 38% in Southern Africa, 18.75% in West Africa, and 17.49% in East Africa. Regional disparities in MCC utilisation can be attributed to several factors. First, variations in study-level characteristics such as study design, sample size, and data collection methods may influence reported utilisation rates. Second, disparities in the availability and accessibility of maternal health care services across the included countries could contribute for the variation. Additionally, sociodemographic factors including education levels, income, cultural beliefs, and geographic accessibility, play a crucial role in shaping MCC utilisation. Addressing these regional disparities requires a multifaceted approach involving strengthening health care systems, addressing sociodemographic determinants, and implementing policies that promote equitable access to maternal and child health services. Future research should prioritise longitudinal and qualitative studies to capture the dynamic and multifaceted nature of MCC utilisation and its underlying determinants across the different regions in SSA.

Significant variations in the level of MCC utilisation were observed across different groups of women. Women aged 25 to 34 showed higher MCC utilisation compared to those aged 35 to 49. Additionally, urban residents were 2.69 times more likely to utilise MCC than rural women. These differences may stem from disparities in health service access, proximity to the health facility, and the availability of quality health services between urban and rural areas [66]. Furthermore, rural residents often face greater challenges accessing health information sources such as health care providers, printed materials, or the internet [67], highlighting the potential barriers to equitable maternal health service utilisation. Moreover, these disparities may reflect differences in health care system performance between urban and rural areas [68]. Such variation in MCC utilisation by residence are critical for health care service planning. The findings of this study are consistent with research conducted in Pakistan [69], underscoring the importance of interventions aimed at enhancing MCC utilisation in rural SSA settings.

The current study also revealed significant disparities in MCC utilisation based on women’s wealth status. Women from richer/richest households had 1.68 times higher odds of utilising MCC compared to those from poorer/poorest households. This finding aligns with the 2015 WHO global report, which noted that births attended by SBAs were 89% in the richest households vs. 43% in the poorest households [70]. Similar findings were observed in Cambodia, where MCC utilisation was higher among wealthier women [59]. Furthermore, the odds of MCC utilisation increased with higher levels of education compared to women with no formal education. This could be attributed to better understanding among educated women regarding the benefits of seeking health care during the perinatal period and their enhanced decision-making power towards maternal health care service utilisation [71,72]. Education is one of the most important factors that affect access to health care services [73]. Studies conducted in India [72,74] and other developing countries [75] have similarly reported these findings. Our study also indicated higher odds of MCC utilisation among employed women compared to unemployed women. Higher educational attainment [76] and employment status [77] are recognised determinants of women’s autonomy in health care decision-making.

In addition to sociodemographic factors, MCC utilisation is influenced by obstetric, medical, husband/partner, and reproductive health characteristics. Although these variables were not included in the current study, research conducted in SSA has identified factors such as distance to health facilities [3,13,26,53,57,78], knowledge of obstetric danger signs [26,52,53], planned pregnancy [21,26,79], birth preparedness and complication readiness [26,53], parity [57], previous history of poor foetal outcome [52], and women’s satisfaction with health service delivery [3] as significant determinants of MCC utilisation. Furthermore, while our study focused on demand side/maternal side factors, supply-side factors such as lack of access, availability, and poor quality could also significantly contribute. Therefore, future studies addressing this gap are recommended.

### Strengths and limitations

This study employed standardised data extraction and study quality assessment tools. We strictly adhered to the PRISMA [24] and MOOSE guidelines [80] throughout the process of searching, study selection, data collection, analysis, and report writing. The robustness of our meta-analysis results was comprehensively evaluated through sensitivity analyses. These analyses demonstrated that the overall findings remained consistent even when individual studies were systematically excluded. Furthermore, the absence of publication bias, as evidenced by the non-significant Egger’s test (*P* > 0.05) and the symmetrical funnel plot, further strengthens the validity of our results. The current study also has several limitations. First, most of the studies included in this review relied on self-reported measures of MCC utilisation and sociodemographic variables, potentially introducing recall bias. Second, our analysis did not encompass supply-side factors beyond location and demographic variables. Third, the cross-sectional nature of the included studies precludes establishing causal relationships between MCC utilisation and independent variables. Additionally, all analyses in this study were unadjusted. Fourth, there was high heterogeneity among the studies included in different meta-analysis, which could be attributed to unexplored study-level covariates in our analysis. This heterogeneity may have impacted the robustness of our findings. To mitigate this, we employed subgroup analysis based on various study-level characteristics and used a random-effects model of analysis. Additionally, we conducted meta-regression and sensitivity analysis. Future research should consider including a broader range of sociodemographic variables and health care system factors to better understand the sources of heterogeneity in MCC utilisation. Furthermore, the inclusion of grey literature in our study aimed to broaden the scope of evidence but may potentially influence the overall findings with data that has not undergone rigorous peer review.

## CONCLUSIONS

Only one-in-five women in SSA utilised complete maternal health services during pregnancy, labour and delivery, and the postpartum period. Older women, those residing in rural areas, with lower wealth status, no formal education, and unemployed status were less likely to utilise MCC. Targeted interventions tailored to sub-regional contexts and addressing significant sociodemographic and economic disparities in MCC utilisation are crucial to improving maternal and child health in SSA. Identifying sub-region-specific factors beyond those studied here is essential to fully understand the determinants of MCC utilisation and to achieve universal health care coverage for maternal health services in SSA. Moreover, future studies should explore supply-side factors associated with MCC utilisation.

## Additional material


Online Supplementary Document
